# Ferroptosis and circular RNAs: new horizons in cancer therapy

**DOI:** 10.17179/excli2026-9663

**Published:** 2026-07-14

**Authors:** Asif Ahmad Bhat, Neelima Kukreti, Muhammad Afzal, Ahsas Goyal, Riya Thapa, Haider Ali, Moyad Shahwan, Waleed Hassan Almalki, Imran Kazmi, Sami I. Alzarea, Sachin Kumar Singh, Kamal Dua, Gaurav Gupta

**Affiliations:** 1 School of Pharmacy, Suresh Gyan Vihar University, Jagatpura, Mahal Road, Jaipur, India; 2 School of Pharmacy, Graphic Era Hill University, Dehradun 248007, India; 3 Department of Pharmaceutical Sciences, Pharmacy Program, Batterjee Medical College, P.O. Box 6231, Jeddah 21442, Saudi Arabia; 4 Institute of Pharmaceutical Research, GLA University, Mathura, U. P., India; 5 Center for Global Health Research, Saveetha Medical College, Saveetha Institute of Medical and Technical Sciences, Saveetha University, India; 6 Department of Pharmacology, Kyrgyz State Medical College, Bishkek, Kyrgyzstan; 7 Department of Clinical Sciences, College of Pharmacy and Health Sciences, Ajman University, Ajman, 346, United Arab Emirates; 8 Centre of Medical and Bio-allied Health Sciences Research, Ajman University, Ajman, Ajman, 346, United Arab Emirates; 9 Department of Pharmacology, College of Pharmacy, Umm Al-Qura University, Makkah, Saudi Arabia; 10 Department of Biochemistry, Faculty of Science, King Abdulaziz University, 21589, Jeddah, Saudi Arabia; 11 Department of Pharmacology, College of Pharmacy, Jouf University, 72341, Sakaka, Al-Jouf, Saudi Arabia; 12 School of Pharmaceutical Sciences, Lovely Professional University, Phagwara 144411, India; 13 Faculty of Health, Australian Research Center in Complementary and Integrative Medicine, University of Technology, Sydney, Ultimo-NSW 2007, Australia; 14 School of Medical and Life Sciences, Sunway University, Sunway, Malaysia; 15 Discipline of Pharmacy, Graduate School of Health, University of Technology, Sydney, Ultimo-NSW 2007, Australia; 16 Uttaranchal Institute of Pharmaceutical Sciences, Uttaranchal University, Dehradun, India

## ⁯Corrigendum

Corrigendum to review article: https://doi.org/10.17179/excli2024-7005

[EXCLI Journal 2024;23:570-599]; PMID: 38887390 ; PMCID: PMC11180955

After publication of the above-cited article, the authors contacted the Editorial Team with the information that “some citations may not be sufficiently aligned with the corresponding statements in the text.” The authors provided the following corrections to the original version of the article:

**Correction 1**

**Location:** Page 572, section “**Understanding circular RNAs**”

**Original text: **“CircRNAs have the ability to affect the onset and course of cancer by altering gene expression and engaging in competition with microRNAs for binding (Bhat et al., 2023d).”

**Corrected text: **“CircRNAs can regulate gene expression by functioning as microRNA sponges (Hansen et al., 2013).”

**References added to reference list:**

Hansen TB, Jensen TI, Clausen BH, Bramsen JB, Finsen B, Damgaard CK, et al. Natural RNA circles function as efficient microRNA sponges. Nature. 2013;495:384-388. https://doi.org/10.1038/nature11993

**References removed from reference list:**

Bhat AA, Gupta G, Afzal O, Kazmi I, Al-Abbasi FA, Altamimi ASA, et al. Neuropharmacological effect of risperidone: From chemistry to medicine. Chem Biol Interact. 2023d;369:110296. doi: 10.1016/j.cbi.2022.110296.

**Correction 2**

**Location:** Page 573, section “**Gastric cancer**”

**Original text:** “Treatment include surgery, chemotherapy, radiation treatment, and targeted medications, depending on the stage of the malignancy (Debela et al., 2021; Zhao et al., 2023).”

**Corrected text: **“Treatment includes surgery, chemotherapy, radiation therapy, and targeted medications, depending on the stage of the malignancy (Debela et al., 2021).”

**References removed from reference list:**

Zhao J, Liu Y, Zhu L, Li J, Liu Y, Luo J, et al. Tumor cell membrane-coated continuous electrochemical sensor for GLUT1 inhibitor screening. J Pharm Anal. 2023;13:673-82. doi: 10.1016/j.jpha.2023.04.015.

**Correction 3**

**Location:** Page 574, section “**Gastric cancer**”

**Original text:** “In Kazakhstan, there has been a declining trend in the incidence and mortality of gastric cancer, with an increase in the frequency of early detection and five-year survival rate (Thapa et al., 2023c).”

**Correction:** This sentence and the corresponding reference are removed from the article.

**References removed from reference list:**

Thapa R, Afzal O, Kumar G, Bhat AA, Almalki WH, Alzarea SI, et al. Unveiling the connection: long-chain non-coding RNAs and critical signaling pathways in breast cancer. Pathol Res Pract. 2023c;249:154736. doi: 10.1016/j.prp.2023.154736.

**Correction 4**

**Location:** Page 574, section “**Gastric cancer**”

**Original text:** “The function of ncRNAs in gastric cancer etiology and progression, such as circRNA, miRNA and lncRNA was also emphasized by the study (Lu et al., 2022; Zhao et al., 2019).”

**Corrected text:** “The roles of ncRNAs, including circRNAs, miRNAs and lncRNAs, in gastric-cancer development and progression were also emphasized (Lu et al., 2022).”

**References removed from reference list:**

Zhao Y, Chen S, Shen F, Long D, Yu T, Wu M, et al. In vitro neutralization of autocrine IL-10 affects Op18/stathmin signaling in non-small cell lung cancer cells. Oncol Rep. 2019;41(1):501-11. doi: 10.3892/or.2018.6795.

**Correction 5**

**Location:** Page 576, section “**Esophageal cancer**”

**Original text:** “Radiation therapy, chemotherapy, and/or surgery are used as treatments (Bhat et al., 2022; Ness-Jensen and Lagergren, 2017).”

**Corrected text:** Treatment of esophageal cancer commonly involves surgery, chemotherapy and radiotherapy, alone or in multimodal combinations (Lagergren et al., 2017).”

**References added to reference list:**

Lagergren J, Smyth E, Cunningham D, Lagergren P. Oesophageal cancer. Lancet. 2017;390:2383-2396. https://doi.org/10.1016/S0140-6736(17)31462-9

**References removed from reference list:**

Ness-Jensen E, Lagergren J. Tobacco smoking, alcohol consumption and gastro-oesophageal reflux disease. Best Pract Res Clin Gastroenterol. 2017;31:501-8. doi: 10.1016/j.bpg.2017.09.004.

**Correction 6**

**Location:** Page 576, section “**Esophageal cancer**”

**Original text:** “Regular tests for persons who are at risk are important because they emphasize the roles that early identification and lifestyle adjustments play in prevention (Bhat et al., 2023e; Miller, 1981).”

**Correction:** This sentence and the corresponding references are removed from the article.

**References removed from reference list:**

Bhat AA, Thapa R, Afzal O, Agrawal N, Almalki WH, Kazmi I, et al. The pyroptotic role of Caspase-3/GSDME signalling pathway among various cancer: A Review. Int J Biol Macromol. 2023e;242(Pt 2):124832. doi: 10.1016/j.ijbiomac.2023.124832.

Miller DG. Principles of early detection of cancer. Cancer. 1981;47(5 Suppl):1142-5. doi: 10.1002/1097-0142(19810301)47:5+<1142::aid-cncr2820471313>3.0.co;2-6.

**Correction 7**

**Location:** Page 577, section “**Lung adenocarcinoma**”

**Original text:** “One common subtype of NSCLC that starts in the peripheral lung tissues is lung adenocarcinoma (Bhat et al., 2023f).”

**Corrected text:** “Lung adenocarcinoma is a major histological subtype of non-small-cell lung cancer (Lemjabbar-Alaoui et al., 2015).”

**References removed from reference list:**

Bhat AA, Thapa R, Goyal A, Subramaniyan V, Kumar D, Gupta S, et al. Curcumin-based nanoformulations as an emerging therapeutic strategy for inflammatory lung diseases. Future Med Chem. 2023f;15:583-6. doi: 10.4155/fmc-2023-0048.

**Correction 8**

**Location: **Page 579, section “**Thyroid cancer**”

**Original text: **“Thyroid cancer comes in two primary forms: follicular and papillary, both of which often have good prognoses (Bhat et al., 2024b).”

**Corrected text: **“Papillary and follicular carcinomas are differentiated forms of thyroid cancer and generally have more favourable outcomes than anaplastic thyroid carcinoma (Grimm, 2022).”

**References removed from reference list:**

Bhat AA, Gupta G, Afzal M, Thapa R, Ali H, Alqahtani SM, et al. Polyphenol-loaded nano-carriers for breast cancer therapy: a comprehensive review. BioNanoScience. 2024b;epub ahead of print. doi: 10.1007/s12668-023-01288-7.

**Correction 9**

**Location:** Page 579, section “**Colorectal cancer**”

**Original text**: “Associated with both hereditary and environmental variables, it frequently develops from benign polyps (Bhat et al., 2023b).”

**Corrected text:** “Colorectal cancer is associated with hereditary and environmental factors and commonly develops through precursor colorectal polyps (Rawla et al., 2019).”

**References removed from reference list:**

Bhat AA, Gilhotra R, Singh Y, Sharma S, Jesus Andreoli Pinto Td, Ferraz HG, et al. Advanced drug-delivery approaches in managing P53-mediated lung diseases remodeling. Nanomedicine. 2023b;18:583-7. doi: 10.2217/nnm-2023-0032.

**Correction 10**

**Location:** Page 579, section “**Colorectal cancer**”

**Original text:** “For successful treatments, early diagnosis through tests such as colonoscopies is essential (Bhat et al., 2023c).”

**Correction:** This sentence and the corresponding reference are removed from the article.

**References removed from reference list:**

Bhat AA, Goyal A, Thapa R, Kazmi I, Alzarea SI, Singh M, et al. Uncovering the complex role of interferon-gamma in suppressing type 2 immunity to cancer. Cytokine. 2023c;171:156376. doi: 10.1016/j.cyto.2023.156376.

**Correction 11**

**Location:** Page 582, section “**Hepatocellular carcinoma**”

**Original text:** “A family of medications known as tyrosine kinase inhibitors targets and inhibits certain tyrosine kinases (Thapa et al., 2023e).”

“Their mechanism of action involves obstructing tyrosine kinase activity, which in turn prevents downstream signaling that advances tumor growth (Bhat et al., 2023a).”

**Corrected text:** “Tyrosine kinase inhibitors block aberrantly activated tyrosine kinases and their downstream oncogenic signalling pathways (Paul and Mukhopadhyay, 2004; Shyam Sunder et al., 2023).”

**References removed from reference list:**

Thapa R, Goyal A, Gupta G, Bhat AA, Singh SK, Subramaniyan V, et al. Recent developments in the role of protocatechuic acid in neurodegenerative disorders. EXCLI J. 2023e;22:595-9. doi: 10.17179/excli2023-5940.

Bhat AA, Afzal O, Agrawal N, Thapa R, Almalki WH, Kazmi I, et al. A comprehensive review on the emerging role of long non-coding RNAs in the regulation of NF-κB signaling in inflammatory lung diseases. Int J Biol Macromol. 2023a;253:126951. doi: 10.1016/j.ijbiomac.2023.126951.

**Correction 12**

**Location:** Page 584, section “**Bladder cancer**”

**Original text:** “Transitional cell carcinoma, which originates in the bladder's innermost lining, is the most prevalent kind of bladder cancer (Thapa et al., 2023f).”

**Corrected text:** “Urothelial carcinoma, historically termed transitional cell carcinoma, is the predominant type of bladder cancer (Farling, 2017).”

**Correction 13**

**Location:** Page 584, section “**Bladder cancer**”

**Original text:** “Urine cytology, imaging investigations, and cystoscopy are just a few of the tests used in the diagnosis process (Thapa et al., 2023a).”

**Correction:** This sentence and the corresponding reference are removed from the article.

**References removed from reference list:**

Thapa R, Afzal O, Alfawaz Altamimi AS, Goyal A, Almalki WH, Alzarea SI, et al. Galangin as an inflammatory response modulator: An updated overview and therapeutic potential. Chem Biol Interact. 2023a;378: 110482. doi: 10.1016/j.cbi.2023.110482.

**Correction 14**

**Location:** Page 585, section “**Bladder cancer**”

**Original text:** “Bladder cancer's epidemiology is typified by its substantial worldwide influence, especially in industrialized nations (Thapa et al., 2022). Globally, bladder cancer was expected to have caused 168,000 deaths and 436,000 new cases in 2018 (Saginala et al., 2020).”

**Corrected text:** “Bladder cancer has a substantial global burden, with approximately 549,000 new cases and 200,000 deaths reported worldwide in 2018 (Saginala et al., 2020).”

**References removed from reference list:**

Thapa R, Gupta G, Dave P, Singh SK, Raizaday A, Almalki WH, et al. Current update on the protective effect of epicatechin in neurodegenerative diseases. EXCLI J. 2022;21:897-903. doi: 10.17179/excli2022-5034.

**Correction 15**

**Location:** Page 585, section “**Bladder cancer**”

**Original text:** “OTU deubiquitinase 1, or OTUB1, is a protein-coding gene that produces an enzyme that is a member of the protease family that contains the ovarian tumor domain (Bhat et al., 2024a). As a deubiquitinating enzyme, OTUB1 contributes to the control of cellular activities that rely on ubiquitin (Rawat et al., 2023).”

**Corrected text:** “OTUB1 is an OTU-family deubiquitinase that regulates protein ubiquitination through canonical deubiquitination and non-canonical inhibition of ubiquitin transfer (Wu et al., 2024).”

**References added to reference list:**

Wu M, Sun L, Song T. OTUB1-mediated inhibition of ubiquitination: a growing list of effectors, multiplex mechanisms, and versatile functions. Front Mol Biosci. 2024;10:1261273. https://doi.org/10.3389/fmolb.2023.1261273

**References removed from reference list:**

Bhat AA, Afzal O, Afzal M, Gupta G, Thapa R, Ali H, et al. MALAT1: A key regulator in lung cancer pathogenesis and therapeutic targeting. Pathol Res Pract. 2024a;253:154991. doi: 10.1016/j.prp.2023.154991.

Rawat S, Gilhotra R, Singh SK, Bhat AA, Ojha A, Dhaundhiyal K, et al. Epigenetics of SARS-CoV2 (COVID-19). In: Gupta G, Oliver BG, Dua K, Ali MK, Dave P (eds): Targeting epigenetics in inflammatory lung diseases (pp 199-208). Singapore: Springer, 2023. doi: 10.1007/978-981-99-4780-5_12.

*Editorial Team*

*EXCLI Journal*

